# Functional outcomes in patient specific instrumentation vs. conventional instrumentation for total knee arthroplasty; a systematic review and meta-analysis of prospective studies

**DOI:** 10.1186/s12891-022-05620-2

**Published:** 2022-07-23

**Authors:** Branavan Rudran, Henry Magill, Nikhil Ponugoti, Andy Williams, Simon Ball

**Affiliations:** 1 Department of Trauma & Orthopaedics, Chelsea and Westminster Hospital, London, SW10 9NH UK; 2 Department of Trauma & Orthopaedics, Fortius Clinic, 17 Fitzhardinge St, London, W1H 6EQ UK

**Keywords:** Total Knee Arthroplasty, Patient-Specific, Meta-analysis

## Abstract

**Background:**

Total Knee Arthroplasty (TKA) is an established surgical option for knee osteoarthritis (OA). There are varying perceptions of the most suitable surgical technique for making bone cuts in TKA. Conventional Instrumentation (CI) uses generic cutting guides (extra- and intra-medullary) for TKA; however, patient specific instrumentation (PSI) has become a popular alternative amongst surgeons.

**Methods:**

A literature search of electronic databases Embase, Medline and registry platform portals was conducted on the 16^th^ May 2021. The search was performed using a predesigned search strategy. Eligible studies were critically appraised for methodological quality. The primary outcome measure was Knee Society Function Score. Functional scores were also collected for the secondary outcome measures: Oxford Knee Score (OKS), Western Ontario and McMaster Universities Arthritis Index (WOMAC), Knee Injury and Osteoarthritis Outcome Score (KOOS) and Visual Analog Scale (VAS) for pain. Review Manager 5.3 was used for all data synthesis and analysis.

**Results:**

There is no conclusive evidence in the literature to suggest that PSI or CI instrumentation is better for functional outcomes. 23 studies were identified for inclusion in this study. Twenty-two studies (18 randomised controlled trials and 4 prospective studies) were included in the meta analysis, with a total of 2277 total knee arthroplasties. There were 1154 PSI TKA and 1123 CI TKA. The majority of outcomes at 3-months, 6-months and 12 show no statistical difference. There was statistical significance at 24 months in favour of PSI group for KSS function (mean difference 4.36, 95% confidence interval 1.83–6.89). The mean difference did not exceed the MCID of 6.4. KSS knee scores demonstrated statistical significance at 24 months (mean difference 2.37, 95% confidence interval (CI) 0.42—4.31), with a MCID of 5.9. WOMAC scores were found to be statistically significant favouring PSI group at 12 months (mean difference -3.47, 95% confidence interval (CI) -6.57- -0.36) and 24 months (mean difference -0.65, 95% confidence interval (CI) -1.28—-0.03), with high level of bias noted in the studies and a MCID of 10.

**Conclusions:**

This meta-analysis of level 1 and level 2 evidence shows there is no clinical difference when comparing PSI and CI KSS function scores for TKA at definitive post operative time points (3 months, 6 months, 12 months and 24 months). Within the secondary outcomes for this study, there was no clinical difference between PSI and CI for TKA. Although there was no clinical difference between PSI and CI for TKA, there was statistical significance noted at 24 months in favour of PSI compared to CI for TKA when considering KSS function, KSS knee scores and WOMAC scores. Studies included in this meta-analysis were of limited cohort size and prospective studies were prone to methodological bias. The current literature is limited and insufficiently robust to make explicit conclusions and therefore further high-powered robust RCTs are required at specific time points.

## Background

Knee osteoarthritis (OA) is a leading cause of global disability [1], with Total Knee Arthroplasty (TKA) accepted as a well-recognised and established therapy [2]. It causes considerable pain and debilitation in the elderly, decreasing their quality of life [3]. There is an overall increase in the incidence of TKA over the past 25 years across all age groups [4]. In the UK, the rates for women who have undergone knee replacements has increased from 43 per 100 000 person years in 1991 to 137 per 100 000 person years in 2006 [5]. There is a predicted increase in the demand of TKA in the USA by 673% by the year 2030 [6]. Although there has been a broadening in surgical options including uni-condylar replacements and tibial osteotomies, total knee replacements remain at the centre of surgical management for OA [7].

Conventional instrumentation (CI) and patient-specific instrumentation (PSI) have been used to aid accurate implant placement [8]. CI for TKA is based on the concept of using intramedullary and extramedullary guides for alignment. Satisfactory post-operative alignment is necessary to achieve good functional outcomes and longevity of the prosthesis [9]. Studies have suggested that both function and survivorship are significantly improved if a post-operative alignment of less than three degrees, in the mechanical axis, is obtained [10, 11].

Patient specific instrumentation (PSI) is custom made using data obtained from computed tomography (CT) or magnetic resonance imaging (MRI) to create bespoke cutting blocks. This imaging also allows pre-operative planning of the best size of prosthesis. In addition, long-leg alignment radiographs enable determination of optimal positioning of the prosthesis. Once optimal positioning and sizing are known, the PSI is built in a 3-dimensional (3D) printer allowing instrumentation creation customised to the patient’s anatomy [12].

If the use of custom-made instrumentation can be proven to improve TKA component positioning it should, in theory, translate to better functional outcomes and prosthetic longevity. A recent meta-analysis by Mannan et al. including literature from 2000 to 2015 revealed eight randomised controlled trials (RCT’s) [13] and concluded that there was no conclusive evidence to support PSI or CI for TKA. Simiarly, a pooled meta-analysis found no difference between PSI and CI at less than one year or at more than one year post surgery [14]. In recent years many high quality studies of level I and level II evidence articles have been published with more varied outcome measures over more substantial periods. This allows for a further scrutination of the functional outcome of PSI and CI at definitive time points. This will allow for a direct comparison between PSI and CI at a given time point.

The present study intends to comprehensively scrutinise the literature to ascertain the result of functional outcomes for PSI compared to CI in TKA, when compared at a specific time point post TKA. In order to comprehensively scrutinise the literature and provide robust clinical recommendations, we have conducted the most current meta-analysis to evaluate the functional outcomes of PSI versus CI for total knee arthroplasty.

## Methods

### Literature search

This systematic review was conducted in accordance with the “Preferred Reporting Items for Systematic reviews and Meta-analyses” (PRISMA) statement [15]. We identified relevant articles through the MEDLINE and Embase databases on the 16^th^ May 2021. A date restriction of January 2004 was specified. The search was performed using MESH terms and free terms for “patient-specific instrumentation” OR “custom-fit” OR “PSI” OR “patient specific instrumentation” AND “total knee arthroplasty” OR “TKA” OR “knee arthroplasty” OR “knee replacement.”

### Eligibility criteria

All randomised controlled trials (RCTs) and prospective comparative studies, where at least one of the selected functional outcomes is reported was considered for inclusion in the study. We adopted a three stage screening process (title screening, abstract screening and full-text screening). All titles, abstracts and full text of articles that were deemed suitable for extraction were retrieved and reviewed independently by two of the co-authors (HM & BR). Consensus was gained amongst all five co-authors where there was disagreement regarding included studies. The study protocol was not registered on PROSPERO in time before the data collection was performed.

### Inclusion criteria

1. Level I and Level II (prospective comparative studies) evidence;
2. Subjects of any country of origin;
3. Patients undergoing primary total knee arthroplasty;
4. A minimum of ten participants in each study arm;

### Exclusion criteria

1. Studies primarily evaluating kinematic or radiographic alignment;
2. Studies of a foreign language were excluded, unless a translation was available;
3. Cutting guide or implant positioning studies;
4. Case reports;

### Outcome measures

The primary outcome measures of interest for this review was Knee Society Score – Function.

The secondary outcome measures of interest for this review were as follows:

1. Knee Society Score (KSS)—Knee
2. Knee Society Score – Global
3. Oxford Knee Score (OKS)
4. WOMAC (Western Ontario and McMaster Universities arthritis Index)
5. Pain score
6. KOOS: KOOS pain, KOOS symptoms, KOOS Sports, KOOS Activities of Daily Living (ADL), KOOS Quality of Life (QOL)

### Searching other resources

The trials register at ClinicalTrials.gov (http://clinicaltrials.gov/) and the World Health Organisation (WHO) International Clinical Trials Registry Platform search portal (http://apps.who.int/trialsearch/) were reviewed for any ongoing or planned trials. Results were screened based on their abstracts and compared against the inclusion and exclusion criteria.

### Data extraction

The data from each study were entered into Microsoft Excel (2007). Data extracted included name of journal, type of patient specific instrumentation used, follow up period, study design and origin, image acquisition, sample size, age of population and outcome measures considered. Data was independently extracted by two co-authors (BR & HM) and verified by another author (NP).

### Data synthesis and statistical analysis

All outcome measures were continuous. A mean difference between PSI and CI groups was determined. The minimal clinically important difference (MCID) was used for all outcomes which were deemed statistically significant. This ensured clinical relevance to the outcomes assessed. The MCID was calculated with linear regression to minimise bias [16].

Review Manager 5.3 was used for all data synthesis and analysis. The “random effects model” was applied if high heterogeneity existed between the compared studies. Final results for each parameter were displayed in a forest plot. Confidence intervals were also displayed. Heterogeneity was formally determined with I^2^ (where 0 to 25% indicates low heterogeneity, 25% to 75% indicates moderate heterogeneity, and > 75% suggests high heterogeneity).

For this meta-analysis, only level 1 and level 2 evidence was used. This ensured improved levels of methodological homogeneity. Clinical heterogeneity – based on characteristics table – similar age group across all studies. Timings of outcomes were delineated to ensure high level of homogeneity.

### Methodological quality assessment

All RCT’s and prospective comparative studies were reviewed independently for quality by two of the co-authors (BR & NP). Where there was any difference in quality assessment, the senior author (SB) made the final decision. The quality of the methods was assessed by the trial quality characteristics and associated risk of bias. For randomised controlled trials, six parameters were used:

1. Randomisation method
2. Allocation Concealment
3. Blinding of participants and personnel
4. Blinding of outcome assessment
5. Incomplete outcome measures
6. Selective reporting bias

Each parameter was labelled as low risk of bias, unclear or high risk of bias. For non-randomised studies, the methodological index for non-randomised studies (MINORS) criteria [17] was used to scrutinise the study against a validated scoring method.

## Results

### Literature search results

**Fig. 1:**
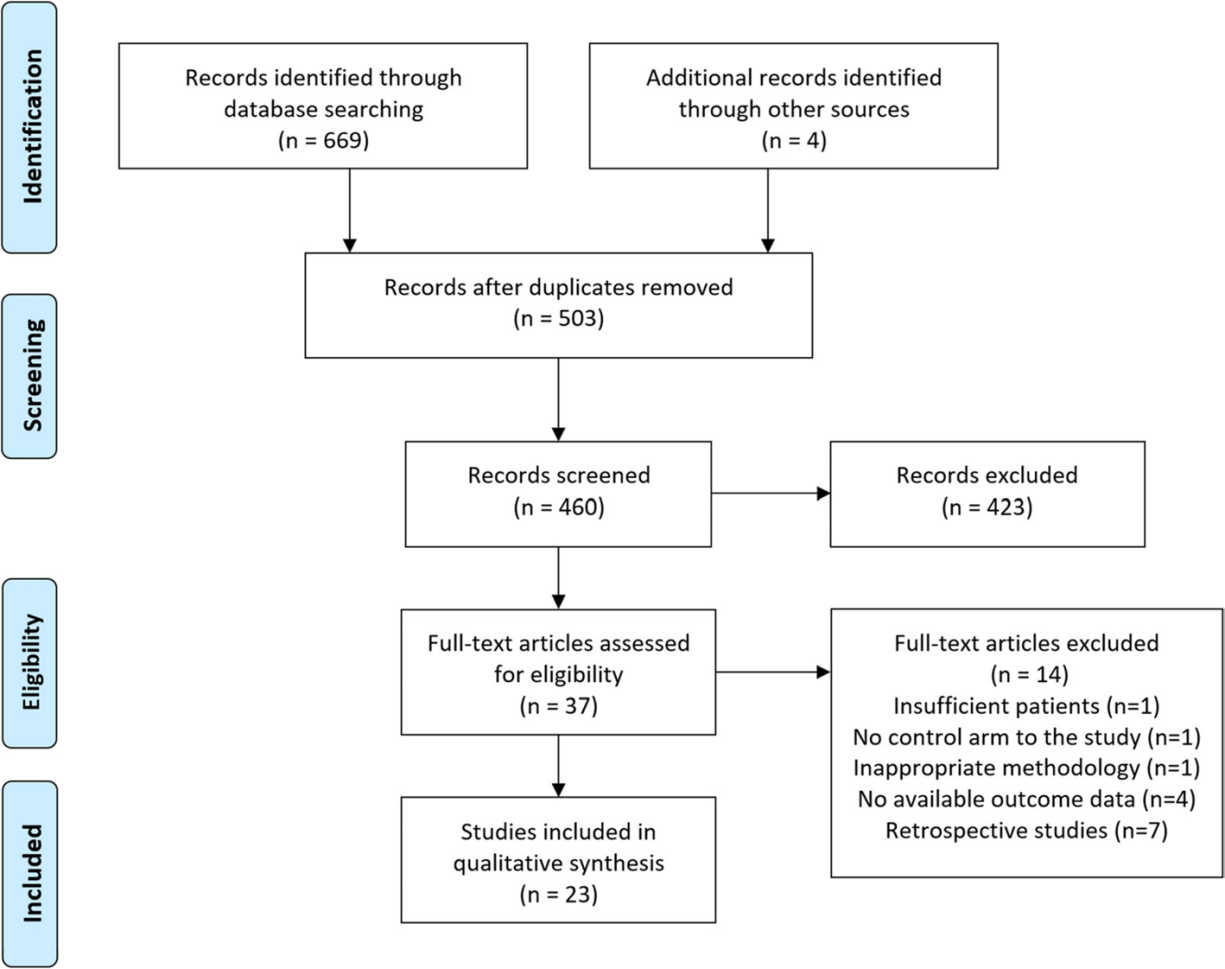
The Preferred Reporting Items for Systematic reviews and Meta-analysis (PRISMA) flow diagram to show study selection

The initial search of the databases yielded 669 results. Four articles were added by identifying suitable articles through other sources. The PRISMA flow diagram for this search is shown in Fig. 1. In total, 23 studies with a total of 2277 total knee arthroplasties were included in the review. There were 1154 PSI TKA and 1123 CI TKA. In the study, 18 RCT’s and 5 prospective studies were included (Fig. 1). 22 studies underwent qualitative review as Moorthy et al. [18] was the only study to report 5 year PROM outcomes.

### Quality assessment

The majority of studies described suitable random sequence generation and randomisation methods. However, there was variable levels of bias due to the methods of allocation concealment across the RCT’s considered. All studies were affected by high risk of bias for the blinding of both participants and personnel. This was partly due to inherent inability to blind the operative team. In addition, patients receiving PSI underwent MRI or CT. This could have led to the patient being aware of the requirement for MRI or CT for a custom made block.

The risk of bias assessment across all the randomised controlled trials of included studies is displayed in Table 1. All non-randomised studies were assessed against the MINORS criteria for comparative studies with a subjective score given out of 24 [17]. A table illustrating the scores is shown in Table 2.

**Table 1:**
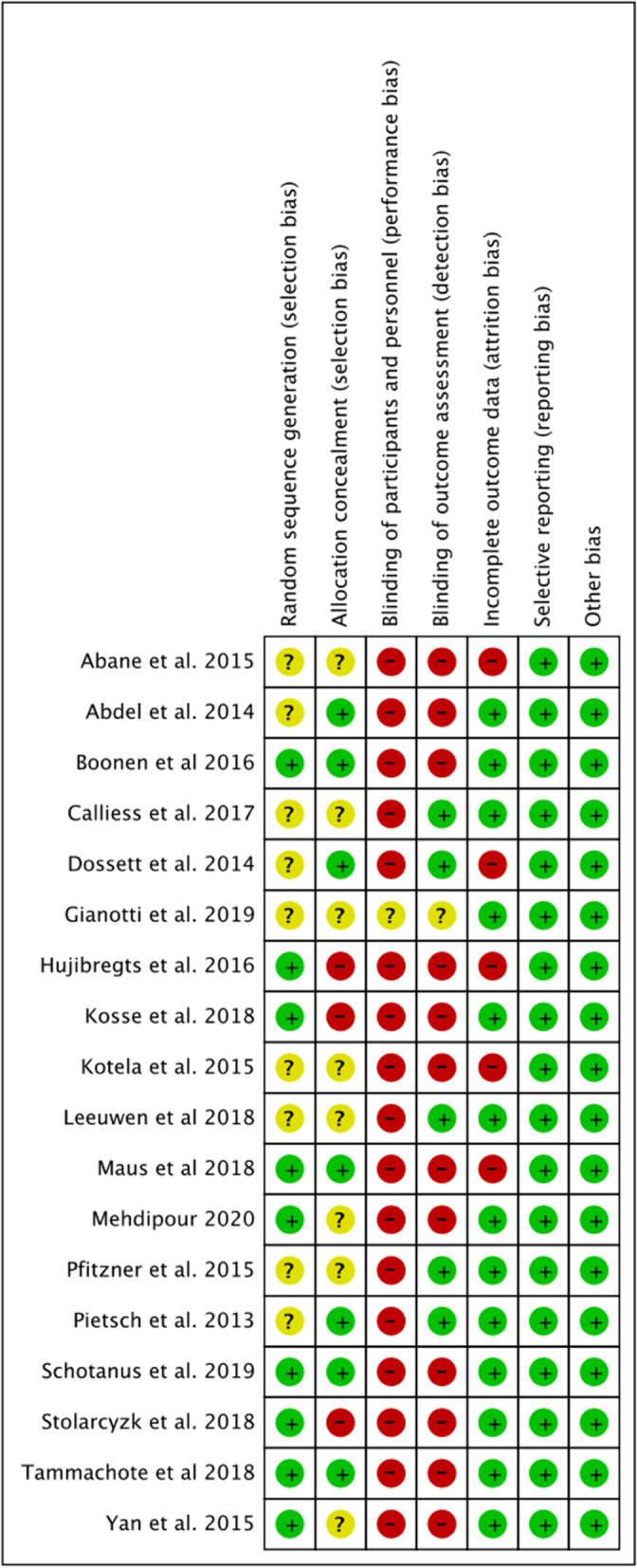
A table displaying the risk of bias for each of the included randomized studies. The colour represents the quality in the each of the domains (Red = High risk, Yellow = uncertain and Green = low risk)

**Table 2:** A table to illustrate the methodological index for non-randomised studies (MINORS) criteria [17]

Paper	Study	MINORS score out of 24
Anderl et al. 2016 [19]	Prospective comparative	18
Chen et al. 2015 [20]	Prospective comparative	20
Yaffe et al. 2014 [21]	Prospective comparative	17
Zhu et al. 2017 [22]	Prospective comparative	17

### Characteristics of studies included

The details of the 23 studies (18 RCTs and 5 prospective studies) included in the systematic review are summarised in Table 3. Twenty-two studies (18 RCTs and 4 prospective studies) were involved in the meta-analysis.

**Table 3:** Characteristics of publications included in the present study

Author	Journal	System used	Follow up	Type of study	Image acquisition	Location	Number of PSI	Number of CI	Mean age
Pietsch et al. 2013 [23]	Knee surgery sports traumatology and Arthroscopy	Materialise	3 months	RCT	MRI	Austria	40	40	71.4(6.6):69.2(9.4)
Pfitzner et al. 2014 [24]	Clinical orthopaedic and related research	Visionaire	3 months	RCT	MRI/CT	Germany	60	30	64.0(54–74): 64.0(54–74)
Yaffe et al. 2014 [21]	Int Journal of Computer assisted radiology and surgery	PSI	6 months	Prospective comparative	MRI	USA	44	40	68.3:64
Abdel et al. 2014 [25]	Clinical orthopaedic and related research	Materialise	3 months	RCT	MRI	France	20	20	71.0(61–81) 71(55–83)
Dossett et al. 2014 [26]	The Bone and Joint Journal	Visionaire	2 years	RCT	MRI	USA	44	44	66.0(7.7): 66.0(8.6)
Abane et al. 2015 [27]	Bone and Joint Journal	Visionaire	3 months	RCT	MRI	France	70	70	67.8(47–84): 70.4(54–83)
Yan et al. 2015 [28]	Knee surgery sports traumatology	Materialise	3 months	RCT	MRI	Hong Kong	30	30	67.5(8.0): 69.5(8.4)
Kotela et al. 2015 [29]	Biomed research international	Signature	12 months	RCT	CT	Poland	49	46	66.1(8.4): 68.6(9.9)
Chen et al. 2015 [20]	Journal of arthroplasty	PSI	2 years	Prospective comparative	MRI	Singapore	29	30	65.0(8.0) 65.0(8.0)
Anderl et al. 2016 [19]	Knee surgery sports traumatology	MyKnee	2 years	Prospective comparative	CT	Austria	114	108	68.7(8.2): 67.7(9.6)
Hujibregts et al. 2016 [30]	The Bone and Joint Journal	Visionaire	1 year	RCT	CT	Holland	69	64	66.7:69
Boonen et al. 2016 [31]	Bone and Joint Journal	Signature	44 months	RCT	MRI	Netherlands	90	90	69.0(8.0):65.0(8.8)
Calliess et al. 2017 [32]	Knee surgery sports traumatology	Stryker Shapematch	12 months	RCT	MRI	Germany	100	100	70(8):67(8)
Zhu et al. 2017 [22]	Knee surgery sports traumatology and Arthroscopy	Trumatch	2 years	Prospective comparative	CT	Singapore	42	48	69.3(7.2):66.8(5.9)
Van Leeuwen et al. 2018 [33]	Acta Orthopaedics	Signature	2 years	RCT	MRI	Norway	44	50	67(8.8):64(6.9)
Stolarczyk et al. 2018 [34]	Clinical and Experimental Biomedcine	Visionaire	3 months	RCT	MRI	Poland	30	30	70.2(5.9):69.6(7.1)
Maus et al. 2018 [35]	Knee surgery sports traumatology and Arthroscopy	Imprint	3 months	RCT	MRI	Germany	59	66	68.1(8.5):71.5(8.1)
Tammachote et al. 2018 [36]	The Journal of Arthroplasty	Visionaire	2 years	RCT	MRI	Thailand	54	54	72.0(7.0):72.0(8.0)
Kosse et al. 2018 [37]	Knee Surgery Sports Traumatology and Arthroscopy	Visionare	12 months	RCT	MRI	Netherlands	21	21	62.7(4.5):63.4(4.2)
Schotanus et al. 2019 [38]	Knee surgery sports traumatology	Signature	5 years	RCT	MRI	Holland	83	80	71.8
Giannotti et al. 2019 [12]	Musculoskeletal surgery	PSI	2 months	RCT	MRI	Italy	20	20	71:73
Mehdipour et al. 2020 [39]	Archives of Bone and Joint Surgery	PSI	2 years	RCT	CT	Iran	12	12	60.3:62.6
Moorthy et al. 2021 [18]	Archives of Orthopaedic and Trauma Surgery	Zimmer PSI	5 years	Prospective comparative	MRI	Singapore	30	30	NR

### Primary outcome

#### *Outcome* 1: Knee Society Score (KSS) – Function

**Fig. 2:**
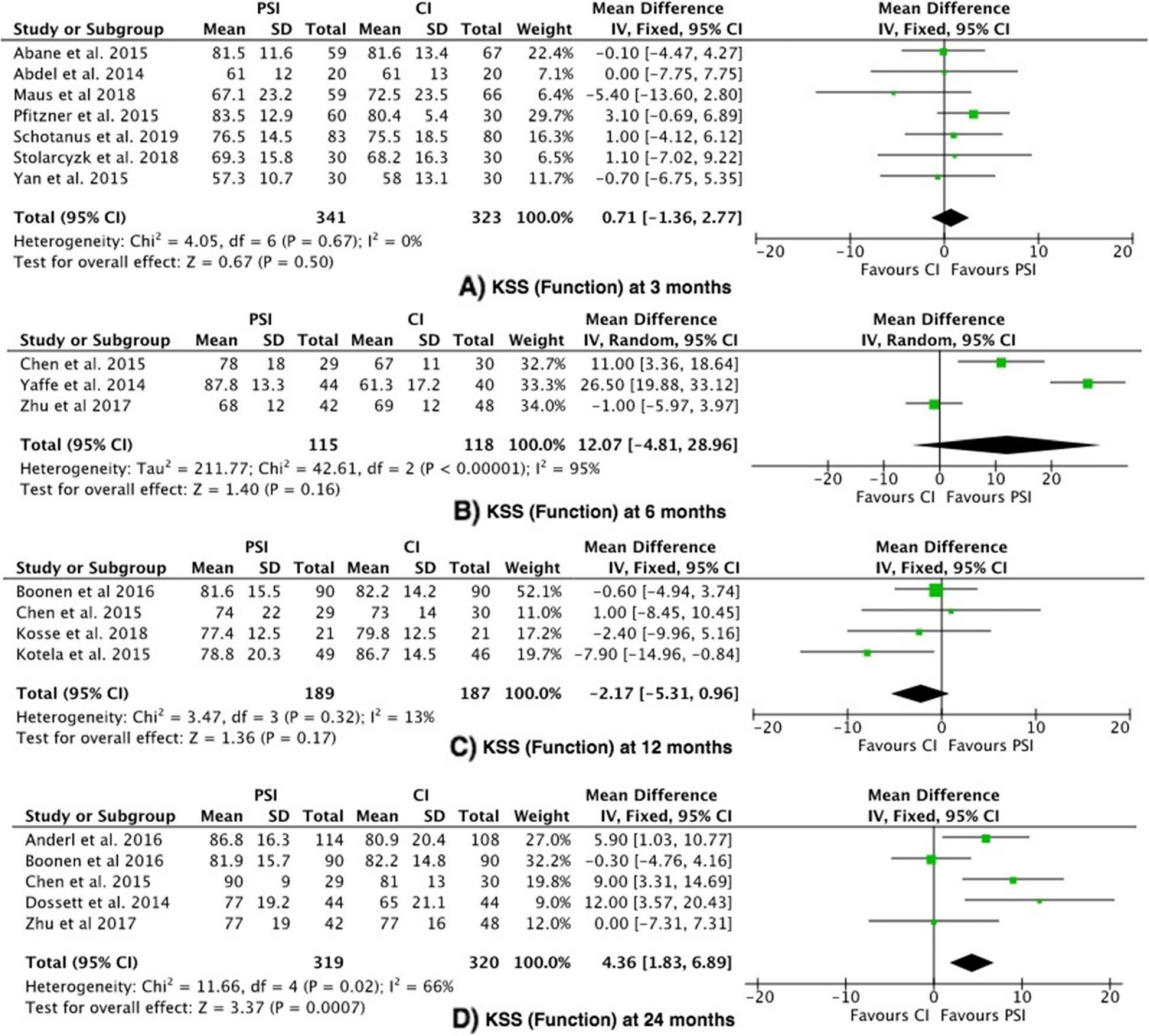
Forest plots of the comparison of KSS (Function) scores at (**A**) 3 Months, (**B**) 6 Months and (**C**) 12 to 24 Months. Abbreviations: CI: Confidence Interval; IV: Independent Variable; M-H: Mantel–Haenszel

The Knee Society Score was reported in 16 studies [19–29, 31, 34, 35, 37, 38]. KSS Functional score was significantly higher in the PSI group (favouring PSI) at 24 months in the 5 studies compared (mean difference 4.36, 95% confidence interval 1.83–6.89), with moderate heterogeneity (I^2^ = 66%) [19, 20, 22, 26, 31]. Three out of the 5 studies reported a statistical significance in the difference between PSI and CI, with regards to KSS function scores [19, 20, 26]. The mean difference (4.36) was less than the MCID (6.4). There was no statistically significant difference observed at 3, 6 or 12 months (Fig. 2).

### Secondary *outcomes*

#### Outcome 2: Knee Society Score (KSS)—Knee

**Fig. 3:**
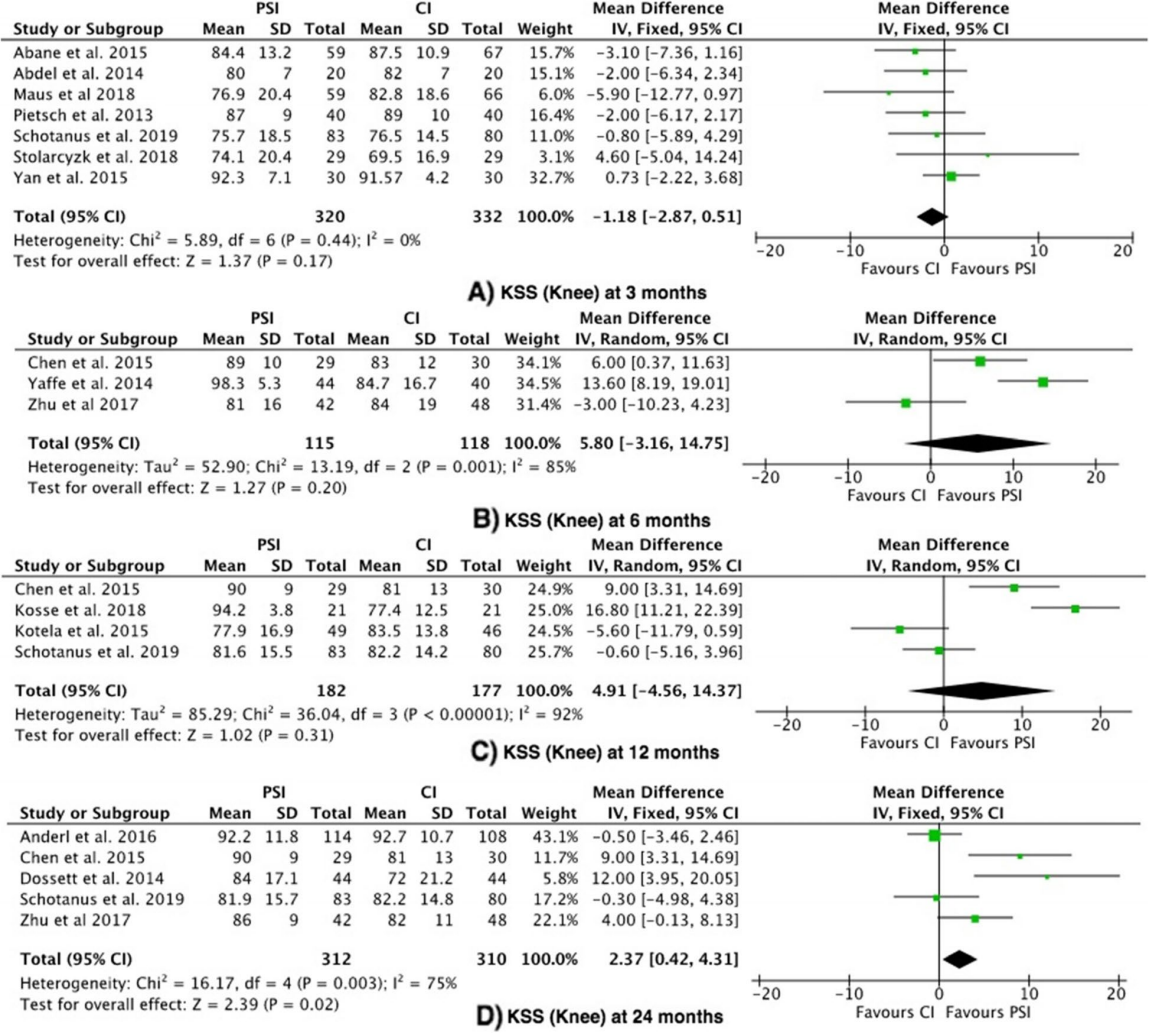
Forest plots of the comparison of KSS (Knee) scores at (**A**) 3 Months, (**B**) 6 Months and (**C**) 12 to 24 Months. Abbreviations: CI: Confidence Interval; IV: Independent Variable; M-H: Mantel–Haenszel

The KSS Knee score was reported in 14 studies [19–23, 25–29, 34, 35, 37, 38]. There was no significant difference observed at 3, 6 or 12 months. The KSS Knee score was significantly higher in the PSI group (favouring PSI) at 24 months in the 5 studies compared (mean difference 2.37, 95% confidence interval (CI) 0.42—4.31), with moderate heterogeneity (I^2^ = 75%) (Fig. 3) [19, 20, 22, 26, 38]. The mean difference (2.37) did not exceed the MCID (5.9).

Four out of the 14 studies were non-randomised prospective studies, which reported MINORS criteria scores ranging from 17–20 out of 24 (Table 22). This shows a high probability of bias in these studies.

#### Outcome 3: Knee Society Score – Global

**Fig. 4:**
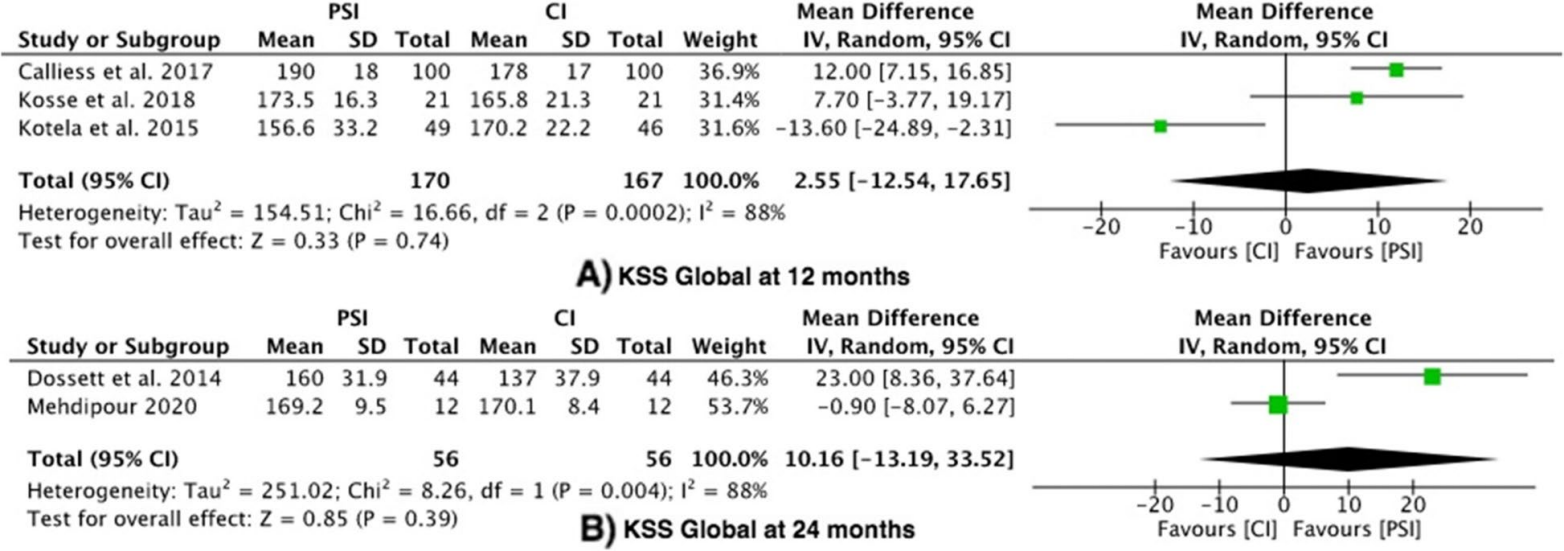
Forest plots of the comparison of KSS (Global) scores at (**A**) 12 Months and (**B**) 24 Months. Abbreviations: CI: Confidence Interval; IV: Independent Variable; M-H: Mantel–Haenszel

Five studies reported the Knee Society Score – global [26, 29, 32, 37, 39], with a high level of heterogeneity (I^2^ -88%). There was no difference between the PSI group and the conventional group at 12 months (mean difference 2.55, 95% confidence interval (CI) -12.54 – 17.65) or 24 months (mean difference 10.16, 95% confidence interval (CI) -13.19 – 33.52) (Fig. 4).

#### Outcome 4: Oxford Knee Score (OKS)

**Fig. 5:**
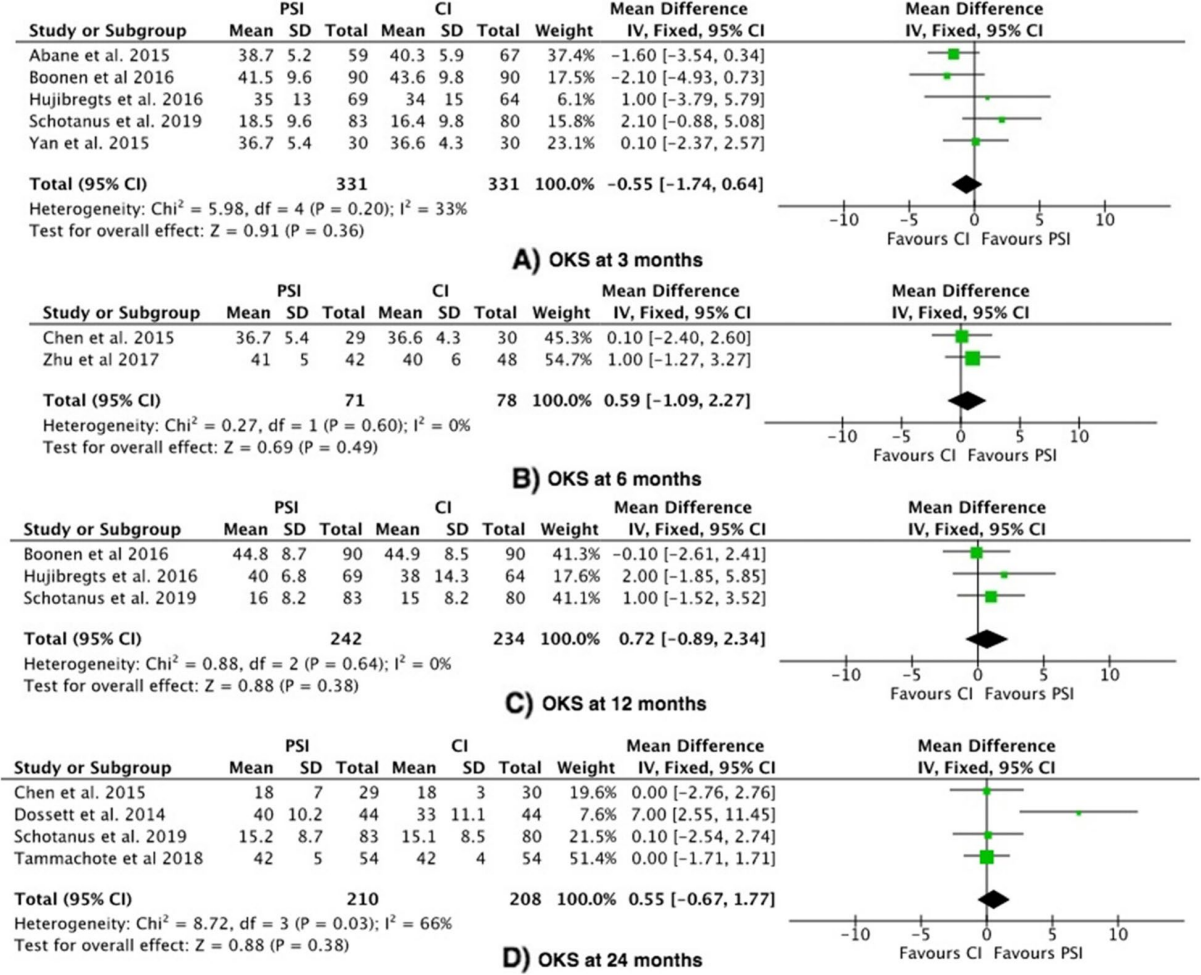
Forest plots of the comparison of OKS scores at (**A**) 3 Months, (**B**) 6 Months, (**C**) 12 Months and (**D**) 24 Months. Abbreviations: CI: Confidence Interval; IV: Independent Variable; M-H: Mantel–Haenszel

The Oxford Knee Score was reported in 9 studies [20, 22, 26–28, 30, 31, 36, 38]. The OKS showed no significant difference between PSI vs. CI at 3 months, 6 months or 12–24 months (Fig. 5) [5].

#### Outcome 5: Western Ontario and McMaster Universities arthritis Index (WOMAC)

**Fig. 6:**
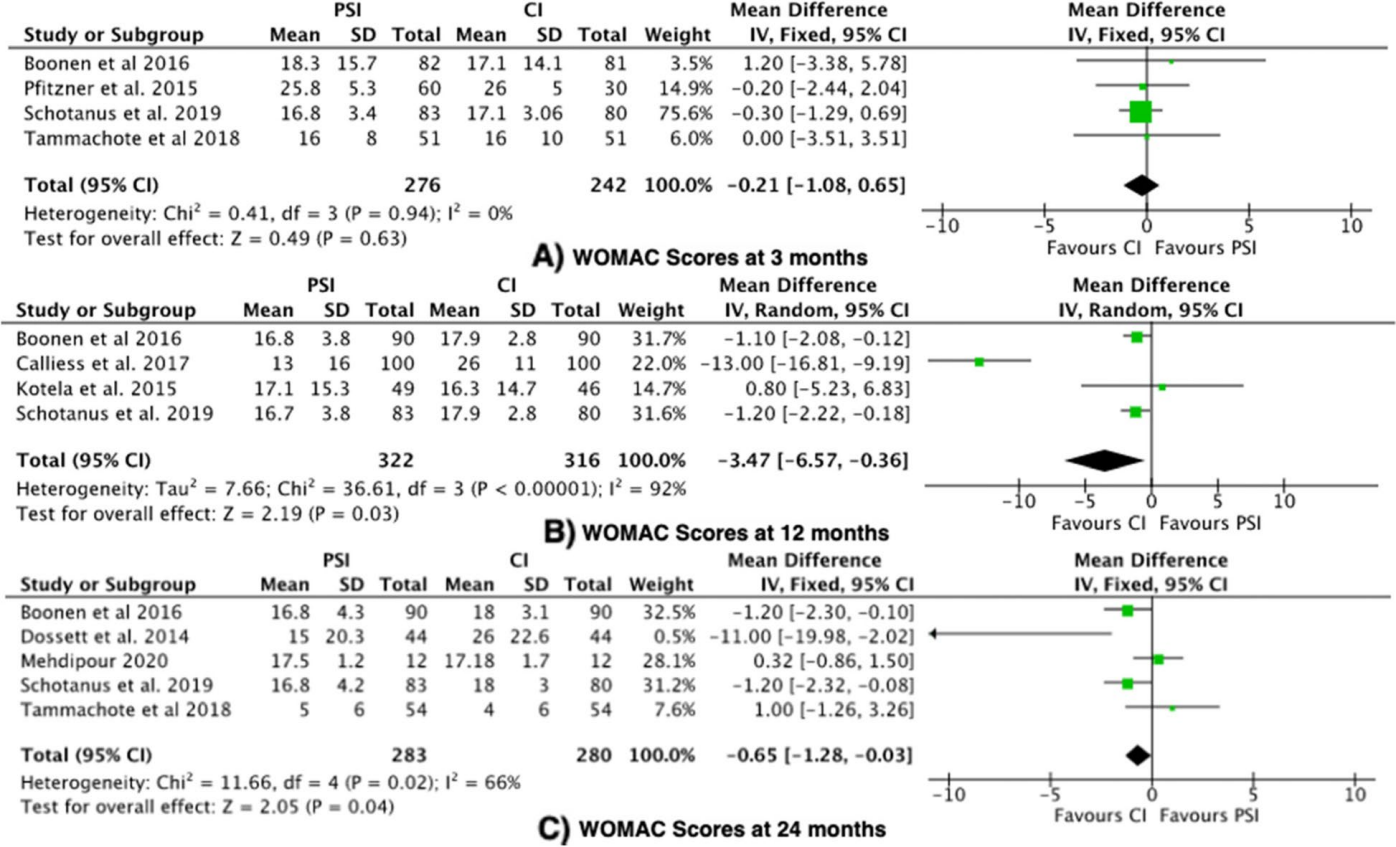
Forest plots of the comparison of WOMAC scores at (**A**) 3 Months, (**B**) 12 Months and (**C**) 24 Months. Abbreviations: CI: Confidence Interval; IV: Independent Variable; M-H: Mantel–Haenszel

The WOMAC Index was reported in 8 studies [24, 26, 29, 31, 32, 36, 38, 39]. The WOMAC score was significantly higher in the CI group (favouring PSI) at 12 months (mean difference -3.47, 95% confidence interval (CI) -6.57—-0.36), with a high level of heterogeneity (I^2^ = 92%). The WOMAC score was significantly higher in the CI group (favouring PSI) at 24 months (mean difference -0.65, 95% confidence interval (CI) -1.28—-0.03), with a moderate level of heterogeneity (I^2^ = 66%) (Fig. 6). Two RCTs showed that the CI group had significantly higher WOMAC scores, where lower scores imply improved function [26, 32]. The mean difference at 12 months and 24 months did not exceed the MCID value of 10 for WOMAC scores.

#### Outcome 6: Pain Score

**Fig. 7:**
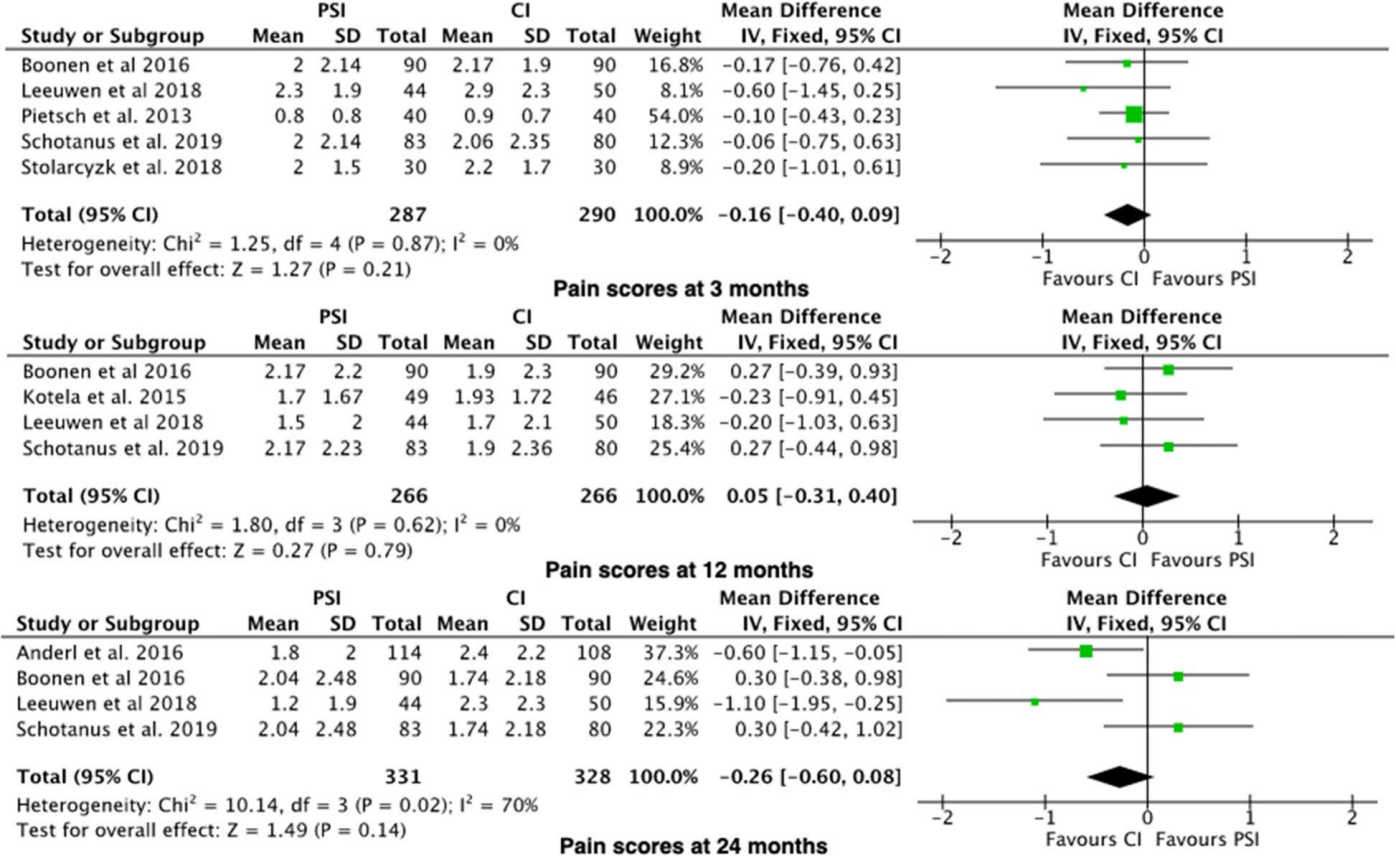
Forest plots of the comparison of pain scores at (**A**) 3 Months, (**B**) 12 Months and (**C**) 24 Months. Abbreviations: CI: Confidence Interval; IV: Independent Variable; M-H: Mantel–Haenszel

The Pain Score was reported in 7 studies [19, 23, 29, 31, 33, 34, 38]. Pain Scores was adjusted proportionally to a 100-point scale. There was no significant difference between PSI vs. CI at 3 months, 6 months or 12–24 months (Fig. 7).

#### Outcome 7: KOOS

**Fig. 8:**
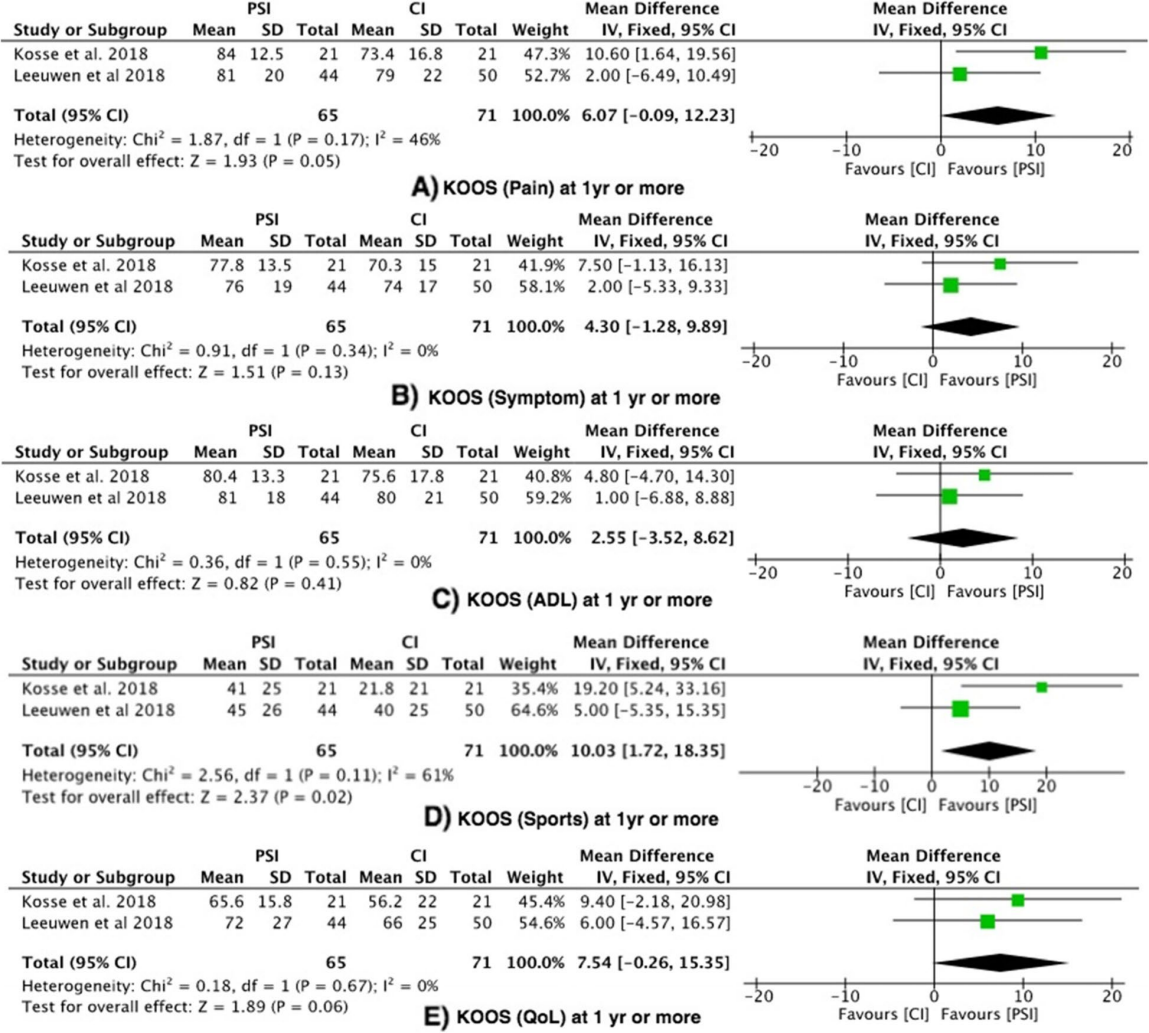
Forest plots of the comparison of KOOS (**A**) Pain, (**B**) Symptom, (**C**) ADL, (**D**) Sports and (**E**) QoL. Abbreviations: CI: Confidence Interval; IV: Independent Variable; M-H: Mantel–Haenszel

Two studies assessed KOOS pain, KOOS symptoms, KOOS ADL, KOOS Sports and KOOS QOL at 3 months and 1 year or more [25, 33] (Fig. 8).

### KOOS pain

There was no significant difference between the PSI group and the conventional group at 3 months or at 1 year or more.

### KOOS symptoms

There was no significant difference between the PSI group and the conventional group at 3 months or at 1 year or more.

### KOOS ADL

There was no significant difference between the PSI group and the conventional group at 3 months or at 1 year or more.

### KOOS Sports

KOOS sports reported no significant difference between the PSI group and the conventional group at 3 months. At 1 year or more, there was a statistically significant difference favouring the PSI group, with moderate heterogeneity (I^2^ = 61%).

### KOOS QOL

There was no significant difference between the PSI group and the conventional group at 3 months or at 1 year or more.

### *Sensitivity* analysis

We performed a sensitivity analysis for all statistically significant results.

KSS knee at 24 months favours PSI. No significant difference between the groups was noted when Chen et al. [20], Dossett et al. [26] and Zhu et al. [22] studies are individually removed. KSS Function at 24 months was found to be statistically significant regardless of individual studies being removed.

WOMAC scores were demonstrated at 12 months and 24 months to favour PSI. When Boonen et al. [31], Dossett et al. *.* [26] and Schotanus et al.  [38] were removed individually, the results were noted to be statistically insignificant. The studies were reviewed individually and deemed suitable for inclusion in the analysis.

## Discussion

This systematic review and meta-analysis included 22 studies (18 RCT’s and 4 prospective studies) to evaluate the functional benefit of TKA using PSI compared to conventional TKA for patients with end stage knee OA. Functional outcomes were assessed at 3 months, 6 months, 12 months and 24 months where published. Only Level 1 and Level 2 evidence were considered for this systematic review to ensure a robust review of the literature.

The primary outcome looked at KSS function scores, which showed PSI was favoured statistically compared to CI at 24 months (mean difference 4.36, 95% confidence interval 1.83–6.89), with no exceedance of the MCID (6.4). Therefore, there was no clinical difference found between PSI and CI. Notably, 3 prospective studies were included in the PSI group at 24 months, which provides an element of bias [19, 20, 22].

For KSS knee scores at 3 months, 6 months and 12 months, there was no statistically significant difference between PSI and CI groups. At 24 months, there was a statistical difference between PSI and CI groups in the 5 studies compared [19, 20, 22, 26, 38], which did not exceed the MCID for KSS knee score. It is noted that 3 out of the 5 studies (*n* = 371) [19, 20, 22] were prospective comparative studies. This results in considerable bias, which is demonstrated by a minors criteria score of 17–20 (Table 2). WOMAC scores were demonstrated to have a statistical difference at 12 months (mean difference -3.47, 95% confidence interval (CI) -6.57—-0.36) and 24 months favouring PSI (mean difference -0.65, 95% confidence interval (CI) -1.28—-0.03), with no exceedance of the MCID value of 10 for WOMAC scores. Two RCTs showed that the CI group had significantly higher WOMAC scores, where lower scores imply improved function [26, 32]. Both RCT’s were randomised adequately, however, allocation concealment and blinding of participants was not performed by Calliess et al. 2017 [32]. This demonstrates substantial bias in the findings found for 12 month WOMAC scores.

Interestingly, KSS global scores demonstrated no statistically significant results at 12 and 24 months. This is similarly shown in the OKS scores at 3,6,12 and 24 months.

A recent meta-analysis by Mannan et al. including literature from 2000 to 2015 revealed eight randomised controlled trials (RCT’s); data were collated to produce a meta-analysis looking at PSI versus CI for functional outcomes [13] at specific time points. Since 2015, many high quality studies of level I and level II evidence articles have been published with more varied outcome measures over more substantial periods [12, 18, 22, 32–39]. A recent meta-analysis by Kizaki et al. [14] conducted pooled analysis of PROMs for PSI vs conventional TKA, which showed PSI did not improve PROM more than TKA. Of note, KSS knee, KSS function, OKS and WOMAC scores were shown to have no statistical difference at less than 1 year or greater than 1 year [14]. These findings corroborate with previous meta-analyses regarding no difference in PROMS irrespective of PSI or conventional TKA. However, the pooled analysis at any time point less than a year and more than a year may result in nuances in the functionality of patients being missed. Our study delineates the time points of post operative PROM to show that there is no clinical difference at definitive time points on the functional outcome.

### Limitations

Although this study demonstrates that there is no clinical difference between PSI and CI in regards to KSS function scores, there are limitations to this paper. The functional outcomes obtained from the studies were from RCTs with limited cohort sizes and qualitative studies. There was a risk of bias in the studies included in this meta-analysis, largely due to inadequacies in the blinding of participants and operating surgeons. The observed heterogeneity is likely due to a wide-range of presenting patient groups and deformities. It may also be attributable to data that is collected from a vast array of surgical centres, operating surgeons of variable experience levels and differing instrument manufacturers. It is appreciated that this paper only looks at functional outcomes in regards to PROMs for comparing PSI to CI. Therefore, this study should provide an impetus for further study on time specific comparisons for PROMs in PSI and CI for TKA.

Recent advances in both navigation and robotic-assisted surgical procedures may also provide a useful tool to assist the surgeon with accuracy of component implantation in TKR. Surgical robotics has been slow to gain acceptance by the majority of practicing surgeons but may hold advantages over conventional knee arthroplasty, or even patient specific instrumentation. Similar RCTs and meta-analysis in this field may ultimately provide these answers.

## Conclusion

The results of this meta-analysis show that when comparing PSI and CI in terms of functional outcomes, the current literature is inadequate to demonstrate superiority of one technique over the other. It is important to appreciate that these findings are limited by the significant level of bias that was observed in the studies reviewed. We appreciate the limitations of the included data, and that PSI may confer benefits in other outcomes not included in this study, including longevity. The literature requires larger, more robust RCT’s on this subject in order to achieve substantial conclusions. In order to establish whether a true functional advantage exists with one instrumentation technique, long-term data is essential.

## Data Availability

All data generated or analysed during this study are included in this published article.

## Abbreviations

OA: Osteoarthritis
TKA: Total Knee Arthroplasty
UK: United Kingdom
USA: United States America
CI: Conventional Instrumentation
PSI: Patient specific instrumentation
CT: Computer Tomography
3D: 3 Dimensional
RCT: Randomised controlled trial
KSS: Knee Society Score
OKS: Oxford Knee Score
WOMAC: Western Ontario and McMaster Universities arthritis index
WHO: World Health Organisation
MINORS: Methodological Index for non-randomised studies
PROM: Patient related outcome measure
MRI: Magnetic resonance imaging
